# Portrait of a lengthy vaccination trajectory in Burkina Faso: from cultural acceptance of vaccines to actual immunization

**DOI:** 10.1186/1472-698X-9-S1-S9

**Published:** 2009-10-14

**Authors:** Marylène Dugas, Eric Dubé, Bocar Kouyaté, Aboubakary Sanou, Gilles Bibeau

**Affiliations:** 1 Dalhousie University, Bioethic department, Halifax, Canada; 2 Université de Montréal, Department of Public Health, Québec, Canada; 3 Centre National de Recherche and de Formation sur le Paludisme, Ouagadougou, Burkina Faso; 4 Université de Montréal, Department of Public Health, Québec, Canada; 5 Department of Anthropology, Université de Montréal, Québec, Canada

## Abstract

**Background:**

The global recognition of vaccination is strongly related to the fact that it has proved in the past able to dramatically reduce the incidence of certain diseases. Nevertheless, reactions regarding the practice of vaccination still vary among communities, affecting the worldwide vaccination coverage. Numerous studies, conducted from varying perspectives, have focused on explaining this active refusal or resistance to vaccination. Although in some cases low immunization coverage has been well explained by active refusal or resistance to vaccination, little is known about the reasons for low coverage where those reactions are absent or play a minor role, especially outside an epidemic context. This study attempts to explain this situation, which is found in the health district of Nouna in Burkina Faso.

**Methods:**

An in-depth ethnographic study was undertaken in the health district of Nouna in an effort to understand, from an anthropological point of view, the logic behind the parental decision-making process regarding the vaccination or non-vaccination of children, in a context where rejection of, and reservations concerning vaccination are not major obstacles.

**Results:**

Three elements emerged from the analysis: the empirical conceptions of childhood diseases, the perceived efficacy of vaccine and the knowledge of appropriate age for vaccination uptake; the gap between the decision-making process and the actual achievement of vaccination; and the vaccination procedure leading to vaccination uptake in the particular context of the health district of Nouna.

**Conclusion:**

The procedures parents must follow in order to obtain vaccination for their children appear complex and constraining, and on certain points discord with the traditional systems of meaning and idioms of distress related to pregnancy, the prevention of childhood diseases and with the cultural matrix shaping decision-making and behaviour. Attention needs to be directed at certain promotional, logistical and structural elements, and at the procedure that must currently be followed to obtain vaccination for a child during routine vaccination sessions, which are currently limiting the active demand for vaccination.

**Abstract in French:**

See the full article online for a translation of this abstract in French.

## Abstract in French

See Additional file 1 for a translation of the abstract to this article in French.

## Background

The global recognition of vaccination is strongly related to the fact that it has proved capable of dramatically reducing the incidence of certain diseases [1-5]. Among many examples are the worldwide eradication of smallpox and of poliomyelitis throughout the West. Vaccination has also led to a drastic reduction of children infected with measles, diphtheria, tetanus and whooping cough [1]. Moreover, vaccines represent a promising avenue in the battles against malaria, Influenza A (subtype H5N1) and the HIV virus [1,6].

Nevertheless, reactions regarding the practice of vaccination still vary among communities, affecting the worldwide vaccination coverage. Nichter [7] and Streefland [8,9] distinguished four basic types of community reactions to vaccination, running in a continuum from refusal to demand: "active refusal or refusal," "passive refusal or resistance," "passive acceptance" and "active demand".

Numerous studies, conducted from varying perspectives, have focused on explaining this active refusal or resistance to vaccination. The social sciences and especially anthropology have raised the question of the biopower, or biopolitics, associated with vaccination in order to explain its rejection [8,10]. Some groups are opposed to vaccination on ethical grounds, claiming that mandatory vaccination represents government interference in what they consider to be a matter of entirely personal choice [11,12]. In the same category are positions citing philosophical or religious beliefs [11] that may limit the introduction of foreign substances into the body [8,10,13-15], or the idea that sickness can only be prevented by an action related to a divinity, an ancestor or some other spiritual entity [13,16,17].

Other studies have been based on psychological behavioural models, using certain elements of the health belief model [18,19] to explain a non-support of vaccination. According to these models, some individuals may be convinced that they run little risk of contracting the diseases targeted by vaccination, or that vaccination represents a greater threat to their health than the diseases themselves [1,11,20]. This reluctance or fear may also be the result of a lack of information concerning the actual effect on the body or the potential side effects in the short, medium and long-term [10,21-23]. Conversely, it may be caused by the bulk of available information, sometimes contradictory, particularly since the advent of domestic access to the Internet, where views for and against vaccination can impede the decision-making process [1,13,21,24].

In African settings more specifically, active refusal has been seen as the result of fear campaigns attempting to undermine national health initiatives, sometimes for a partisan purpose, or alternatively under the influence of religious or moral African leaders who, again, see vaccination as contravening their fundamental doctrinal principles [8,10,11,13-15]. Very often these groups believe, or encourage the population to believe, that vaccination activities are a cover for a form of birth control by contraceptive injection, that the AIDS virus can be transmitted via the vaccination procedure, or simply that it is forbidden by their beliefs. Other studies evoked the organisational problems of the Expanded Program on Immunization (EPI) and its poor health care quality, including overestimated cost of vaccination records, incoherence in the follow-up of vaccination and disruption in the cold chain [25]. Passive refusal, according to the results of available studies in African settings, occurs on a more individual basis, each person having their own reasons for refusing vaccination, including fear of side effects, insufficient time to attend vaccination sessions or simply lack of motivation [7-9].

In Burkina Faso, campaigns designed to raise awareness about the importance of vaccination seem to have had positive results regarding the acceptance level of vaccination, notably during the last vaccination campaign against the meningitis epidemic of 2002. According to the report produced by the World Health Organisation/European Commission's Humanitarian aid Office (WHO/ECHO), all modern and traditional means of communication were used to encourage communities to accept vaccination, and the population responded very positively. In Ouahigouya, a city located in the north of Burkina Faso, the city's only private radio station was mobilized for the occasion. Messages were broadcast in French and Moré, reminding citizens of the importance of vaccination and giving information about the places and dates of upcoming vaccination sessions. The awareness campaign received additional support from traditional chiefs and religious leaders, and the promotion was a success, resulting in major participation in the vaccination program in that epidemic context [26].

Although low immunization coverage has been well explained by active refusal or resistance to vaccination, little is known about the reasons for low coverage, especially outside an epidemic context, where those reactions are absent or play a minor role. The question now is: how can one explain the low rates of vaccination attendance outside of epidemic contexts in parts of the world where general levels of opposition to the practice are quite low, where one find mostly passive acceptance or demand and where the various obstacles inducing active or passive refusal are almost absent? This study attempts to explain this situation, which is found in the health district of Nouna in Burkina Faso.

## Methods

In order to answer this question, an in-depth ethnographic study was undertaken in the health district of Nouna as part of the ETUVAC (ETUde sur la VACcination) strategy aiming at improving immunization coverage among children aged 0 to 11 months old. It was performed in an effort to understand, from an anthropological point of view, the logic behind the parental decision-making process regarding the vaccination or non-vaccination of children, in a context where rejection of, and reservations concerning vaccination are not major obstacles.

The health district of Nouna is situated in the northwest of Burkina Faso, around 300 km from the capital of Ouagadougou. This region is inhabited by five principal ethnic groups (Marka, Bwaba, Samo, Mossi and Peulh). The population, close to two-thirds of whom are illiterate, is essentially rural and agricultural, with subsistence agriculture predominating. There are 27 peripheral health posts (Centres de Santé et de Promotion Sociale - CSPS) in the health district of Nouna that cover a population of about 300,000 inhabitants. The health professionals of the CSPS are assisted in their tasks by Village Health Officers (VHOs), who are voluntary workers chosen in each village to transmit information about upcoming immunization sessions between the CSPS and their own village.

Regarding the vaccination coverage of the health district of Nouna, the administrative data derived from the vaccination registers of various CSPS have established the effective vaccination coverage of children from 0 to 11 months in 2002, prior to the ETUVAC strategy. It revealed that only 84% of this cohort received the Bacillus Calmette-Guerin vaccine (BCG - tuberculosis), 54% received the third dose of Diphtheria-Tetanus-Pertussis vaccine (DTP3), 57% received the Measles Vaccine (MV) and 51% received the Anti-Amaril Vaccine (AAV-yellow fever). It was, at that time, well below the national objectives outlined by the WHO and the EPI, which are set at 91% for BCG and 80% for AAV, MV and DTP3 vaccines [27].

The conceptual framework underlying this study in medical anthropology is built around three key concepts: systems of meaning, idioms of distress and cultural matrices. The "Systems of meaning" focuses on the meanings of illness, prevention, as well as the forces that govern access to, and demand for, prevention through vaccination [28]. "Idioms of distress" refer to the suffering experienced by parents as the result of childhood diseases and deaths. These narratives of suffering attempt to circumscribe the uncertainty generated by the possibility of seeing a disease become serious and by the difficulties of effectively treating it or preventing it. These idioms are also present in the discussions surrounding the advantages and inconveniences of having children vaccinated. "Cultural matrices" encompass the moral premises, collective values and beliefs that shape systems of meaning and idioms of distress. Parental behaviour (reluctance, refusal, acceptance) regarding vaccination can be understood in terms of the cultural matrix on which the parents depend to give meaning to the events of their daily lives. It is important to note, however, that the emphasis placed here on the realm of meaning in no way implies that the concrete behaviour of parents is unaffected by the conditions (material, social, political) that impact day after day on the lives of often illiterate families whose priority is the struggle for survival in particularly difficult economic circumstances [29].

In order to collect the data on those subjects, focus groups were performed to gather general data among several categories of individuals taking into account ethnicity, gender and parental experience. General data concerning health, disease, and immunisation was collected with the participation of individuals issued from four ethnic groups: the Marka, Bwaba, Mossi and Peulh. The participants were selected from among four average size villages in the health district of Nouna: Solimana (a Marka village), Kemena (a Bwaba village), Dennissa-Mossi (a Mossi village) and Tebere (a Peulh village). The populations of these villages range from 544 inhabitants (Tebere) to 2,052 inhabitants (Kemena), and they are all situated between 5 and 10 km from the nearest CSPS. Sixteen focus groups (four groups in each one of the four villages) composed of three people, were performed. In each village (ethnic group), the four focus group were composed of either 1 - three primiparous mothers, 2 - three multiparous mothers (three or more children), 3 - three grandmothers or 4 - three fathers (or grandfathers). The different participants were not necessarily related.

After the focus groups, semi-moderated interviews were performed with eight families, which included eight heads of households and their respective wives (and co-wives when applicable). Issues raised at the focus groups by the other participants were discussed in greater depth. In order to understand the factors influencing vaccination uptakes, these semi-moderated meetings were held with two different categories of families: 1 - Four families in which all the children had been adequately vaccinated, and 2 - Four families in which the children's vaccination records showed numerous gaps. Each of these families was chosen among the same four villages and ethnic groups, but with different participants than for the focus groups, for a total of eight independently interviewed families: two Marka families, two Bwaba families, two Mossi families and two Peulh families.

The data were collected with the assistance of four professional translators specially trained by Nouna's health research center (Centre de recherche en santé de Nouna - CRSN) to lead the semi-moderated interviews according to a preset interview scheme and to moderate focus group discussions in the local dialect. Each one was chosen for their experience and linguistic knowledge related to one of the four communities. Simultaneous translation was chosen to ease the interaction between the main investigators, who did not master all the dialects, and the participants. The data was written down on paper for further analysis. The traditional semiology and nosology issues related to diseases were taken into account and discussed with the translators prior to the interviews and focus groups. The closest biomedical translation was then used to ease the analysis. For instance, a fever is usually called "hot body" by the participants, convulsions are called "bird disease" and malaria is called "cool/humidity disease". These biomedical translations are commonly used by researchers and generally accepted for the purpose of the different studies performed in the district.

As a first step, the acceptability of the vaccines was discussed with the participants. They were then questioned on their conceptualisation of the "normal" development of a child to understand how their systems of cultural representations influence their views concerning the onset of various diseases. The goal was therefore the discovery of which diseases, or which symptoms perceived by the community as diseases, are placed in the category of "normal" or inevitable childhood diseases, in other words, the diseases that all children contract at least once during their childhood and that are more or less unavoidable. The participants were also asked which among these diseases, or among other diseases not necessarily considered normal, did they perceive as belonging to the category of diseases that could cause a child's death - fatal diseases, some of which can or should be prevented by a prophylactic treatment such as vaccination, enhancing the demand for vaccination. They finally were asked to talk about the adverse effects that they knew vaccines to cause. Parental knowledge regarding the target age categories of children by the EPI was also discerned. The cultural matrix determining the role of the father, the mother and the grandparents in the decision-making process concerning vaccination, and about the gap between these determinants and actual behaviours, as reflected in the presence or absence of the mother at advertised vaccination sessions, was also discussed. Finally, the trajectory that must be taken, the different steps needed in order to be able to have their child vaccinated, and the burden that this can imply, were investigated.

## Results

### The acceptance of vaccination

The reaction encountered among the communities questioned about vaccination was general acceptance. During the meetings with the communities, all the interviewed individuals knew about the regular vaccination programs and the periodical visits to their villages by medical staff for the purposes of vaccinating the children, and all said to be in favour of vaccination, i.e. they all wish to obtain the various offered vaccines, either for themselves or for their children:

"Vaccination is a good thing to prevent disease, even for elderly people. It's well thought of [here in the village], especially since everyone in the village was vaccinated against an epidemic in the 1970s." *Marka father*

One after the other, in every village, each participant clearly expressed his/her acceptance of vaccination. There even seemed to be a certain community demand for it as a preventive method, particularly in the case of an epidemic, as expressed by the remarks of the Marka father just quoted, who proudly showed his vaccination scar and encouraged others to show theirs as proof of what he was saying.

In what follows will be presented three elements that emerged from the analysis that might help to explain why, in a context of acceptance of, and demand for vaccination, rather than rejection, is encountered such a low rate of vaccination coverage. These are: the empirical conceptions of childhood diseases, the perceived efficacy of vaccine and the knowledge of appropriate age for vaccination uptake; the gap between the decision-making process and the actual achievement of vaccination; and the vaccination procedure leading to vaccination uptake in the particular context of the health district of Nouna.

### Childhood diseases

The diseases (or symptoms considered as diseases) most frequently mentioned by the participants were all considered to be both normal/inevitable and potentially fatal. They are, in frequency order: fever, diarrhoea, chickenpox, cough, convulsions, bodily pains (head, stomach), malaria and vomiting (Table 1). The three main diseases/symptoms named (fever, diarrhoea and head pain) were also mentioned as being common side effects of vaccination, with diarrhoea and fever being the most frequently cited side effects of vaccination. They are followed by minor injury to the arm (swelling, soreness, bruising).

**Table 1:** Frequency of mentioned diseases/symptoms.

Disease/symptoms	Considered Normal/inevitable (n)	Considered potentially fatal (n)	Considered as side effects (n)
Fever	10	5	8
Diarrhea	9	5	3
Stomach/head pains	6	3	1
Cough	8	4	-
Malaria	6	1	-
Chickenpox	4	8	-
Convulsions	3	8	-
Vomiting	3	3	-
Meningitis	-	3	-

Frequency table of the mentioned diseases/symptoms. 1: considered normal/inevitable; 2: considered potentially fatal; 3: considered as possible side effects, by the participant of the focus groups (n = total of participants who mentioned the disease/symptom).

No consistent differences between the said normal diseases and those known to be fatal were found in their conceptualisation of the known bulk of childhood diseases. Among the symptoms or diseases enumerated by the participants, meningitis (or stiff neck) was the only one identified (on three occasions) as a fatal disease that is neither normal nor inevitable among children, and which it is consequently particularly important to prevent through vaccination.

Finally, when the participants were asked to tell at what age they thought children should be vaccinated to avoid these diseases, the majority replied "between the age of one and five".

### The decision-making process

The data showed that among the communities studied, making the decision to accept, or not, the immunization of a child is mainly the role of the head of the household. According to the majority of the participants interviewed, the decision is made sometimes by the father alone, sometimes by the father's father (the paternal grandfather) and sometimes by the father and mother or grandmother together:

"The father does make the decision for the vaccination." *Mossi grandmother*

"The father makes the decisions in general." Primiparous *Marka mother*

"If there is a grandfather, the father loses control, he won't be informed." *Primiparous Marka mother*

"The father asks the grandfather. They decide together. They also ask the grandmother." *Marka father*

"It belongs to the father. For girls, it last until their wedding." *Peulh grandmother*

"Paternal grandfather and father." *Multiparous Peulh mother*

"The father makes the decision. When he doesn't make the decision quickly, the mother can try to convince him." *Bwaba father*

"The grandmother might be involved in the decision, particularly for the protection of the child." *Bwaba grandmother*

"The father makes the decisions." *Primiparous Bwaba mother*

"Everyone, but especially the mother." *Primiparous Mossi mother*

Among the different communities, the Bwaba and Mossi mothers can be involved in the decision-making. In the Marka and Peulh families, the grandfather seemed more present in the decision-making process. The grandmother was cited by the Bwaba and Marka as being part of the decision-making process.

As it has been said, the decision makers are generally in favour of vaccination given the fact that the vaccines are offered free of charge, and because, according to them, it keeps children healthy and prevents disease. However, it was also clearly stated during the focus groups and interviews that sometimes, despite the fathers' decision to vaccinate the children, the mothers do not always bring children to the immunization sessions offered.

"If the mother doesn't want to have her child vaccinated, she won't do it." *Primiparous Marka mother*

"The fathers make the decision, but if they don't take care, some women won't get their child vaccinated." *Marka father*

"It is the father [who accepts], it is the mothers who refuse and some women leaves and don't get their child vaccinated." *Mossi grandmother*

The only participants who maintained that the women always entirely respect their husbands' decision regarding vaccination were members of the Peulh community, in which the submission of women to their husbands is culturally more marked.

When the participants were asked about why, according to them, some mothers did not always attend vaccination sessions with their children, they gave three frequent types of answers. The first was that families were not informed "because they live too far away and didn't hear the public crier" (Multiparous Bwaba mother). The second concerned individuals who do not vaccinate their children because they "are travelling, people who are away or get there too late" (Bwaba grandmother). Finally, the third category encompassed a number of reasons all related to the mothers' behaviour:

"The mothers are afraid for their children when they hear the other children crying." *Multiparous Marka mother*

"Often, the mothers are busy working and after it's too late and it's over." *Primiparous Bwaba mother*

Either they said it was because they are afraid for their children, or they do not wish to or cannot interrupt their work in the house. The latter reason is the one most frequently evoked by various participants in the study.

### The vaccination procedure

The last factor that emerged from the meetings with the selected participants is structural in nature and concerns in particular the problems associated with the procedure leading to acquisition of the vaccination booklet. The booklet can be obtained free of charge during one of the free antenatal consultations (ANC) offered at the CSPS. This booklet is required for the vaccinations offered in villages by vaccination officers during routine vaccination sessions, which is not the case during special vaccination campaigns; in these cases, the families are given a vaccination card. Having this booklet at the moment of the vaccination session seemed problematic for many participants:

"Vaccination is not available to mothers who don't have the child's booklet. So they have to return home or go and get the booklet from the CSPS." *Multiparous Bwaba mother*

**Figure 1:**
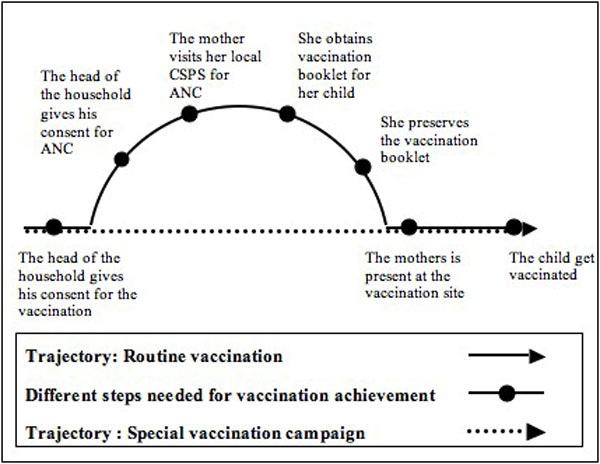
**Comparison of vaccination trajectories**. Comparison diagram between routine vaccination activities and special vaccination campaigns.

The additional procedure involved in getting a child vaccinated during routine vaccination sessions, i.e. acquiring the booklet and having it for the session, implies four stages that have been revealed by and discussed with the participants. First, 1 - the head of the household must give his consent for an ANC. Then, 2 - the mother must visit her local CSPS for an ANC. 3 - During this consultation, she must receive a free vaccination booklet for her child. Finally, 4 - she must preserve the vaccination booklet in good condition until the birth of her child and afterwards for a period ranging from 11 months to two years. Figure 1 illustrates the difference between the procedure for obtaining vaccinations during routine vaccination sessions and during special vaccination campaigns.

This multi-stage procedure seemed problematic for the families interviewed, especially for the group of families in which the children's vaccination records showed numerous gaps. They expressed their concern that the procedure was far more complex than simply showing up at vaccination sessions or accepting vaccinations during the door-to-door visits made during special vaccination programs.

#### 1 - Consent by the head of the household for the ANC

In the first place, most of the heads of households spoken to who have neglected their children's vaccination revealed that they do not believe in the necessity of an ANC during their wives' pregnancies. They consider motherhood to be a natural process that should not be medicalized. Many of the women revealed that either they or someone they knew had been refused vaccination for their child because they had not attended an ANC and therefore did not possess a vaccination booklet.

In addition, some heads of households interviewed were under the impression that they had to pay for an ANC. On questioning them more closely, it became clear that most of the first ANCs had taken place when the mother had gone to the CSPS because of illness, and that she had indeed been required to pay for a medical record (distinct from her child's vaccination booklet) and for the consultation, which was not considered to be a simple antenatal visit, but a full medical consultation, and was thus not offered free of charge.

#### 2 - Mother's trip to a CSPS for an ANC

According to the participants, even in cases where the head of the household has given his consent for an ANC, the distance the mother has to travel (between 5 and 10 km for the communities interviewed), often by foot, to reach the nearest CSPS and the time thus wasted discourages some mothers from making the trip, especially if their pregnancy is already far advanced.

Also, all the interviewed families whose children had a relatively good vaccination status and who possessed the most vaccination booklets were related to the VHO. The VHO is the principal source of information about health activities scheduled to take place in the village and about requirements for obtaining vaccinations, such as the acquisition of a vaccination booklet during an ANC.

It seems, according to the data, that these individuals have a greater influence over members of their own family than over the rest of the villagers, who frequently complained that they were only informed at the last minute about vaccination sessions being held in their village or about the need to procure a vaccination booklet during an ANC.

"There's not enough information before the vaccination, the public crier should come more often before the vaccination sessions. Those who live alone or very far away don't get the information in time." *Multiparous Bwaba mother*

#### 3 - Acquisition of the child's vaccination booklet

Some of the questioned participants did not seem aware that the vaccination booklet required by vaccination officers is available to their child free of charge. Some were convinced they had to pay for the child's booklet, others even claimed to have refused to pay for booklets that had been offered them.

"It's free [vaccination], there's no problem, but you have to pay from 25 to 100 CFA francs for the vaccination booklet, and this is why lots of people don't have their children vaccinated." *Multiparous Bwaba mother*

"Vaccinations are good, but they give you fever and its costs money - to get fever! Also, they charge 25 CFA francs for the weighing and 150 CFA francs for the booklet." *Multiparous Peulh mother*

"At a month old my child was vaccinated five times, but the booklet cost me 225 CFA francs." *Primiparous Mossi mother*

No data exists to assess exactly why they were charged for those vaccination booklets. It might be due to confusion between those booklets and maternity booklets or regular medical booklets that do have to be paid for, or it might be the price to get a lost or destroyed booklet replaced.

#### 4 - Preservation of the vaccination booklet

Preserving the vaccination booklet in good condition and not losing it seems to represent a major problem for the mothers interviewed. A number of mothers complained about how difficult it is to preserve a paper booklet, since for most of them there is no spot in the hut where they can place such a document out of reach of rain, termites and small children. As the mothers were asked to show their vaccination booklets and cards in order to check their children's vaccination status, it was possible to observe the condition of the booklets. Many of them were indeed gnawed by termites or soaked by rain, and were as a result unusable or unreadable.

## Discussion

Most of the individuals who participated in focus groups or interviews and who failed to have their children completely vaccinated did not do so because they do not care for their children's health or because they strictly reject vaccination. The comments reported in the first part of the results reveal the parents' real preoccupation with their children's health and the general acceptance of vaccines. But, as it will be discussed now, some obstacles linked to their conceptualisation of childhood diseases and the appropriate age for vaccination, decision-making processes and vaccination procedure still hamper the achievement of good vaccination coverage in this particular area.

### Childhood diseases

The common denominator between all symptoms/diseases and side effects of vaccination that have been frequently named by participants is the ease with which they can be recognized and identified, even by people who do not have a strong biomedical knowledge or who do not have access to sophisticated antigen tests. They are all distinct and highly noticeable expressions of an abnormal condition of the body. Also, the repetition of the experience of an illness in the immediate environment is the primary means for most of those individuals to acquire biomedical and traditional medical knowledge [17]. This may be the source of this frequent association between the normal childhood diseases/symptoms which are frequently observed, like fever or diarrhoea, and the fatal diseases. For instance, as children regularly show signs of fever or diarrhoea prior to death (due in part to the great burden of malaria and gastrointestinal parasites) and considering that the exact cause of death is usually not known because of the lack of biomedical screening tests or medical consultation, it is understood by the family that the fever, or diarrhoea *per se *is actually the main cause of death [17]. As all children get these symptoms at least once in their life, the disease is spontaneously considered to be "normal" and, as some children unfortunately die while presenting those common symptoms, the disease is also commonly considered as "potentially fatal".

The importance assigned by participants to the symptoms of diarrhoea and fever may constitute a problem in the effort to increase vaccination coverage rates. It could also negatively influence the perception of the vaccination itself. Numerous participants claimed to be in favour of vaccination on the grounds that it "keeps children healthy," "prevents disease" or "treats children." In fact, four of the vaccines used in the EPI target a single disease (BCG, AAV, MV, OPV-Oral Polio Vaccine); only one vaccine targets three diseases (DTP). Given the relatively limited effect of vaccines, their empirical efficiency may prove disappointing when a fully vaccinated child falls victim to diarrhoea or fever, especially if these conditions are the result either of the side effects of vaccination, of a less severe disease or a disease for which there is no available vaccine, such as malaria.

This empirical conception of childhood diseases may play a significant role in the low rates of vaccination coverage existing in the health district studied, particularly for vaccination programs taking place outside an epidemic context. It has been noted elsewhere that the shift from the use of vaccination to control epidemics in developing countries to its use in preventing epidemics that have not yet occurred can lead to a marked reduction in general optimism and in the demand for vaccination [13]. The spectacular effects of the former are not there to maintain the popularity of routine vaccination among the targeted populations. The difficulty lies in trying to convince the communities that vaccination does prevent some childhood diseases when, empirically, 1 - fever and diarrhoea keep on affecting children after vaccination, and 2 - fever and diarrhoea can be among the side effects of vaccination.

Also, the fact that a majority of participants thought the appropriate age to get their child vaccinated was over one year old might have a negative impact on the rate of vaccination coverage, especially when it comes to calculating the valid coverage rate regarding acquisition of all the vaccines available to children of less than a year. To be considered valid the Polio3, BCG, DTP3, VAA, and VAR vaccines must be received before the age of one. This finding is consistent with other studies in Sub-Saharan Africa where it is estimated that only 50% of the children living in that part of the world are vaccinated in the year after their birth [30].

### The decision-making process

Because of the gender difference in the decision-making process, which is more pronounced among the Peulh community compared to the other groups studied, the head of the household is usually the main target of vaccination awareness campaigns. But the dual decision-making process, revealed by the data, involving first the father, who gives his consent, and then the mother, who for different reasons decides otherwise, demonstrates that actual vaccination awareness campaigns that still focus mainly on fathers need to be shifted towards the mothers to raise their awareness of the importance of vaccination for their child's health.

It is important to note here that the grandmother (mainly the paternal grandmother), who enjoys a higher social status than the mother and who possesses a certain influence over her grandchildren, especially in the Bwaba and Marka communities, could serve as a valuable promotional co-agent. It remains to be seen to what extent the grandmother could also be mobilized, in the husband's absence, to help, persuade and remind the mothers of the neighbourhood to set aside their other duties for a short while in order to take their children to a vaccination session.

### The vaccination procedure

During special vaccination campaigns, it is necessary to raise awareness among heads of households in order to obtain tacit authorization for the child's vaccination. The door-to-door approach generally employed during such campaigns allows for an easier contact with a maximum number of families. For routine vaccination sessions, even greater effort must be put into raising awareness among village authorities and mothers as has just been discussed, in order to ensure that the mothers not only show up at the vaccination site but also that they obtain, during an ANC, the child's vaccination booklet and bring it with them in good condition.

#### 1 - Consent by the head of the household for the ANC

The mandatory institutionalization of pregnancy and birth and the obligation to attend an ANC in order to obtain the child's vaccination booklet appeared to be a major obstacle to the vaccination of children in the villages concerned.

In sub-Saharan Africa, we estimate that only 44% of all women receive a full ANC, so we can assume that around 56% of mothers risk not having the vaccination booklet in time for their child [31-33].

What is important here is that the various communities are being asked not only to accept vaccination for their newborns and to be present at vaccination sessions as in the case of special vaccination campaigns, but also - in addition to all the difficulties outlined above - to accept the ANC, thus considerably increasing the cases of parental negligence and passive refusal with regard to vaccination.

#### 2 - Mother's trip to a CSPS for an ANC

There is no doubt that the fatigue engendered by a long walk and the time taken away from the mothers' household duties have a negative impact on campaigns aimed at raising awareness about the importance of ANCs, and by extension on campaigns designed to raise awareness about the need to have children vaccinated. Access to health care thus becomes a major obstacle in the achievement of vaccination, despite the good intentions of health authorities who, having pronounced themselves in favour of an advanced vaccination strategy, ensure that vaccination sessions are generally held in the centre of each village.

Asking mothers to make the trip to the nearest CSPS during their pregnancy undermines the increased access to services offered by advanced vaccination strategies and, as a result, potentially reduces vaccination coverage. The advanced vaccination strategy of the EPI has contributed considerably to the increase in vaccination coverage in Burkina Faso since the early 1980s [34,35]. In that context, it might be worthwhile to introduce a similar strategy for ANCs, thus ensuring that mothers acquire the child's vaccination booklet and at the same occasion receive the anti-tetanus vaccine, which is, after all, the child's very first vaccine that should be received while still in the mother's womb.

#### 3 - Acquisition of the child's vaccination booklet

The comments of the participants stating that they have to pay for the child's vaccination booklet may result from some families confusing the vaccination booklet, which is free, with the regular medical or maternity booklets, or with subsequent replacement booklets, which they are asked to pay for. Another anthropological study in the West African context exploring the population perception of the EPI exposed similar findings, linking the booklet acquisition problems and the actual vaccination achievement [25]. Boa's study in the semi-rural department of Bouna in the Northwest of the Ivory Coast revealed that the over billing of the vaccination booklet was leading to passive refusal of vaccination [25]. More research is needed here to determine if this is the case or not in the health district of Nouna, Whether it is or not, however, it is vital that the various communities be informed that the vaccination booklet is entirely free when requested during an ANC, so as to ensure that no more mothers are refused vaccination for their child owing to the lack of a booklet.

#### 4 - Preservation of the vaccination booklet

Mothers who lose their child's booklet or who are presenting a booklet in bad condition to a vaccination officer are sometimes refused vaccination for their child, thus adding a new group of unvaccinated children to those who are refused vaccination because their mother has not previously procured a booklet during an ANC.

Vaccination booklets are in principle intended as reference documents that the parents can consult to keep track of their child's vaccination status. However, they include no pictograms and are therefore of no use to most parents, who are illiterate and cannot read the information it contains. The fact that parents use the booklet only on the occasion of vaccination sessions increases the risk that it will get lost, be destroyed or becomes unusable. According to Dabiré [36] who worked on this issue in Ouagadougou, Burkina Faso, mothers, because of their illiteracy, do not appreciate the importance of the booklet, and end up losing it. The loss of booklets has also been evoked by researchers in other contexts, including by Tremblay [37] in Haiti and Millimouno [15] in Guinea, where both have indicated that the booklet was easily and frequently lost. Each has also established that those losses have contributed to the non-vaccination of children.

The sense of shame experienced by mothers as a result of the loss or poor state of their booklets, which was also observed during this study when they showed them to the researchers for verification, is undeniable, for it is in some sense a reflection of their poverty and illiteracy. But when this feeling is triggered by public reproach from the vaccination officer during vaccination sessions, and when a mother is harshly criticized in front of other mothers for failure to keep her booklet in good condition, this can create a genuine aversion to the whole vaccination process. A mother singled out in this way may simply decide to stop attending vaccination sessions, and her name will be added to the already far too lengthy list of parents who fail to have their children vaccinated.

For those reasons, it might be worth evaluating the cost-effectiveness of introducing changes that would enable mothers to keep track of their children's vaccination status, such as producing an illustrated booklet, facilitating preservation of the booklets by coating them with plastic, or adopting a centralized tracking system.

## Conclusion

The procedures parents must follow in order to obtain vaccination for their children appear complex and constraining, and on certain points discord with the traditional systems of meaning and idioms of distress related to pregnancy, the prevention of childhood diseases and with the cultural matrix shaping decision-making and behaviour. Promotional and awareness campaigns conducted among these populations have proved fruitful. Attention now needs to be directed at certain promotional, logistical and structural elements, and at the procedure that must currently be followed to obtain vaccination for a child during routine vaccination sessions, which are currently limiting the active demand for vaccination.

It must be acknowledged, however, that the results and conclusion that have been presented here are very specific to this particular setting, the health district of Nouna, and thus can not be applied generally to all situations in which are found low levels of coverage coupled with low levels of opposition to immunisation. This constitutes a limit to this study. However, the research question might still hold valid in the search to find culturally and contextually sensitive answers and solutions for the benefit of other populations experiencing a similar situation.

## List of abbreviations used

AAV: Anti-Amaril vaccine; ANC: Antenatal consultations; BCG: Bacillus Calmette-Guerin; CRSN: Centre de Recherche en Santé de Nouna (Nouna's health research centre); CSPS: Centres de santé et promotion sociale (peripheral health posts); DTP: Diphteria-Tetatus Petussis Vaccine; EPI: Expanded Program on Immunization; ETU-VAC: Étude sur la vaccination (Vaccination study); MV: Measles vaccine; VHO: Village health officials; WHO/ECHO: World Health Organisation/European Commission's Humanitarian aid Office.

## Competing interests

The authors declare that they have no competing interests

## Authors' contributions

MD participated in the design of the study, carried out the field studies, analysed the data and drafted the manuscript. ED carried out the field studies, contributed in the analyses of the data and drafted the manuscript. BK conceived the study, participated in its design and coordination and critically revised the draft. AS participated in the design of the study and critically revised the draft. GB conceived the study, participated in its design and coordination and helped to draft the manuscript. All authors read and approved the final manuscript.

## Supplementary Material

Additional file 1 Abstract in French. Click here for file
