# Supplementary material for: Portrait of a lengthy vaccination trajectory in Burkina Faso: from cultural acceptance of vaccines to actual immunization
Source: BMC Int Health Hum Rights. 2009 Oct 14;9(Suppl 1):S9. doi: 10.1186/1472-698X-9-S1-S9 (PMC3226241; doi:10.1186/1472-698X-9-S1-S9)
Supplement: Additional file 1 — Abstract in French. [file 1472-698X-9-S1-S9-S1.pdf]

Portrait d’un long parcours vaccinal au Burkina Faso : de l’acceptation culturelle à l’obtention des vaccins.

Marylène Dugas, Eric Dubé, Bocar Kouyaté, Aboubakary Sanou, Gilles Bibeau

Résumé

Problématique

La grande popularité de la vaccination est sans doute liée au fait qu’elle a su démontrer par le passé qu’elle pouvait réduire de façon spectaculaire l’incidence des maladies évitables par la vaccination. Néanmoins, les positions face à la vaccination varient parmi les différentes communautés, affectant les taux de couverture vaccinale dans le monde. Diverses études, réalisées selon différentes perspectives, ont traité du phénomène de refus ou résistance active à la vaccination. Bien que, dans certains cas, la faible couverture vaccinale ait été bien expliquée par le refus ou la résistance active à la vaccination, on en sait peu sur les raisons de la faible couverture dans des régions où ces réactions sont absentes ou jouent un rôle mineur, en particulier en dehors d'un contexte épidémique. Cette étude tente d'expliquer cette situation dans le district sanitaire de Nouna au Burkina Faso.

Méthodes

Un travail approfondi de recherche ethnographique a été entrepris dans le district sanitaire de Nouna pour comprendre, d’un point de vue anthropologique, la logique qui organise le processus de prise de décision de la part des parents de faire vacciner ou non

leurs enfants dans un contexte où le refus ou les réticences ne sont pas des barrières majeures à la vaccination.

Résultats

Trois éléments se sont dégagés de l'analyse : les conceptions empiriques des maladies de l'enfant, la perception de l'efficacité du vaccin et la connaissance de l'âge approprié pour la vaccination ; la différence entre la prise de décision en faveur de la vaccination et l'obtention réelle de la vaccination ; et, le parcours vaccinal menant à l'obtention de la vaccination dans le district sanitaire de Nouna.

Conclusions

Les procédures que les parents doivent suivre en vue d'obtenir la vaccination de leurs enfants paraissent trop complexes et contraignantes et, à certains égards, détonnent avec les systèmes de sens locaux et les idiomes de la détresse liée à la prévention des maladies de l'enfance et à la grossesse. Elles se présentent en rupture avec la matrice culturelle affectant le processus de prise de décision et les comportements en lien avec la vaccination. L'attention doit maintenant être portée sur différents éléments de promotion du vaccin, de logistique de distribution, de structure et aux procédures exigées pour l'obtention de la vaccination lors des sessions de vaccination de routine, lesquels limite la demande active de la vaccination.
